# Translation and validation of the Dutch language version of the CDC Symptom Inventory for assessment of Chronic Fatigue Syndrome (CFS)

**DOI:** 10.1186/1478-7954-4-12

**Published:** 2006-10-18

**Authors:** Ruud CW Vermeulen

**Affiliations:** 1 CFS and Pain Research Center Amsterdam, Amsterdam, The Netherlands

## Abstract

**Background:**

In a study by Wagner et al., the CDC Symptom Inventory was validated in a population selected from the inhabitants of a city in the USA, and proofed reliable for the assessment of the accompanying symptoms of CFS. The Dutch translation of the CDC Symptom Inventory is compared to the original and the psychometric properties are presented for patients in a tertiary care setting.

**Methods:**

One hundred thirty-nine consecutive patients who visited the CFS Center Amsterdam for the first time were asked to complete the CDC Symptom Inventory in the Dutch Language Version (DLV) together with the usual set of questionnaires. Sixty-one patients had Chronic Fatigue (CF) and 78 patients fulfilled the criteria for CFS. Forty-three healthy accompanying persons completed the CDC Symptom Inventory DLV, the Physical Functioning scale of the Medical Outcome Survey Short Form-36 DLV, and the Fatigue and Concentration scales of the Checklist Individual Strength (CIS-20).

**Results:**

The healthy controls group contained fewer women and was overall older than the patient groups. The influence of gender on the CDC Symptom Inventory DLV was significant but the effect of age was not. The Dutch version had a good internal consistency and convergent validity. The results were comparable to the original English version, but the sex-related difference needs further study.

**Conclusion:**

The Dutch version of the CDC Symptom Inventory is a reliable tool for the assessment of the secondary criteria for CFS. The results show that it is comparable to the outcome of studies in English speaking countries.

## Background

Chronic Fatigue Syndrome (CFS) is a disabling state that was defined by a working group in 1994 [1]. The main components of the definition are fatigue that is not related to exercise and not relieved by rest, and eight accompanying symptoms, of which four must be present. The CFS is incapacitating, with a serious reduction in daily activity.

Several self-rating scales for the presence and severity of fatigue were developed. Of these, the Multidimensional Fatigue Inventory (MFI-20) [2] and the Checklist Individual Strength (CIS-20) [3] were selected by an international CFS study group [4]. The same group advised the Medical Outcomes Survey Short-Form-36 (SF-36) as the tool for the assessment of functional impairment. For the presence of the accompanying symptoms of CFS, a symptom checklist developed by the Centers for Disease Control and Prevention was suggested. The MFI-20 and the CIS-20 were developed in the Dutch language and validated. The SF-36 was translated and validated [5]. The CDC Symptom Inventory was validated for the English-speaking countries [6]. The translation in Dutch was considered necessary for comparison of data in CFS research in the Netherlands and other countries. The objective of the present study was to translate the CDC Symptom Inventory and to validate it for the Dutch speaking population.

## Methods

The participants in the study were all patients who attended the CFS Center Amsterdam for the first time for diagnosis and treatment of chronic fatigue from August 2005 to August 2006 and their healthy accompanying persons. No investigations were added to the standard diagnostic protocol for new patients in the Center and the accompanying persons were asked to complete the questionnaires only. Accompanying persons who reported fatigue of one month or more, or were ever identified with medical or psychiatric conditions exclusionary for CFS were excluded from the study. All participants gave informed consent for the use of their data for this study.

The CIS-20 is a 20-item self-report instrument that measures 4 dimensions of fatigue: fatigue, concentration, impaired motivation and impaired activity. For clinical assessment of fatigue we used the fatigue and concentration subscales. The CDC Symptom Inventory DLV was used for the assessment of the presence of additional symptoms and their severity. Symptoms were rated as suggested by Wagner et al. [6]. We calculated the CDC Symptom Inventory DLV Total Score, the CDC Symptom Inventory DLV Short Form, the CDC Symptom Inventory DLV Case Definition Score and the CDC Symptom Inventory DLV Other Symptoms score as indicated by the authors. The severity of physical impairment was measured with the physical functioning subscale of the SF-36. All healthy controls completed a list of questions about health, medical interventions in the past and drug use. A physical checkup and laboratory data according to the recommendations of Fukuda et al. [1] were obtained from all patients.

### Translation

The English version of the CDC Symptom Inventory was translated into Dutch by a native Dutch speaker fluent in English. The translation was presented to 4 native Dutch speakers for problems in acceptance and comprehension of the questionnaire content or the phrasing. The provisional Dutch version was translated back into English by a native English speaker fluent in Dutch.

### Statistical analysis

We evaluated the internal consistency of the CDC Symptom Inventory DLV by performing a reliability analysis based on the model of averaging the inter-item correlation.

In the male and female groups the convergent validity between the CDC Symptom Inventory DLV, the CIS-20 and the physical score of the SF-36 was tested by the calculation of Pearson's correlation coefficient. Construct validity by one-way of variance analysis and Bonferroni post-hoc group comparisons were used to compare the CDC Symptom Inventory DLV scores, the CIS-20 scores and the SF-36 score across the three groups. To determine whether there were differences between CDC groups, a series of multivariate analyses of covariance was conducted, with physical impairment, fatigue or concentration as dependant variables and with gender and age as covariates.

All statistical analyses were carried out using the Statistical Package for the Social Sciences (SPSS version 14.0).

## Results

Forty-three healthy controls completed the questionnaires. Sixty-one patients fulfilled the Fukuda criteria for chronic fatigue (CF-group) and 78 patients those for chronic fatigue syndrome (CFS-group) (Table 1). The difference between men and women was significant for all scores (Table 2) (Student's *t*-test *P *< 0.001). The differences were partially caused by the age distributions that were not the same for men and women in the Control, CF and CFS groups. The difference of the age distribution between the 3 groups was significant for men and women together (Jonckheere-Terpstra Test: *P *= 0.013). When tested separately the age differences between the 3 groups were neither different for women (Jonckheere-Terpstra Test: *P *= 0.287) nor for men (Jonckheere-Terpstra Test: *P *= 0.177). Analysis of covariance, controlling for age and gender, demonstrated significant differences between the three groups [Λ = .165, *F *(6,174) = 84.7, *p *< .000]. The analysis for men and women was done separately.

**Table 1:** Characteristics by subject classification (n = 182).

Classification	Number		Age (Mean ± SD)	
	Female	Male	Female	Male

CFS	61	17	39 (13)	44 (12)
CF	47	14	37 (12)	42 (10)
Controls	19	24	47 (16)	48 (12)

CFS: patients with chronic fatigue syndrome, CF: patients with chronic fatigue, not meeting the criteria for CFS, and healthy controls without fatigue complaints.

**Table 2:** Descriptive data of the CDC Symptom Inventory DLV, Medical outcomes Survey Short-Form 36 and Checklist Individual Health scores.

CDC Symptom Inventory Scores	Mean	SD	Min	Max
Female (n = 127)				
Total	83.74	45.15	0	201
Short Form	45.78	25.13	0	96
CDC Case Definition	47.61	24.89	0	104
Other Symptoms	36.07	24.02	0	105
SF-36 Physical	57.13	25.21	0	100
CIS Fatigue	47.60	12.00	8	56
CIS Concentration	25.40	8.65	5	35
Male (n = 55)				
Total	48.79	48.21	0	158
Short Form	29.89	29.70	0	82
CDC Case Definition	26.96	25.71	0	85
Other Symptoms	21.79	24.58	0	92
SF-36 Physical	74.73	25.77	5	100
CIS Fatigue	34.94	18.90	8	56
CIS Concentration	18.09	10.59	5	35

### Reliability analysis

Reliability analyses showed good internal consistency for the CDC Symptom Inventory Total score with a Cronbach's alpha coefficient of 0.86 for women and 0.91 for men. Cronbach's alpha coefficients were 0.85 (women) and 0.91 (men) for the Symptom Inventory Short Form, 0.79 and 0.85 for the Symptom Inventory Case Definition score, and 0.75 and 0.82 for the Symptom Inventory Other Symptoms score.

Table 2 shows the descriptive data for the Total, Short Form, Case Definition and the Other Symptoms scores. Table 3 shows the corrected item-total correlations (product terms) of the symptoms for the Total score, the Short Form and the Case Definition.

**Table 3:** Corrected item to total correlations for the CDC Symptom Inventory DLV Total score, the Symptom Inventory Short Form score and the Symptom Inventory Case Definition score.

Symptom	Corrected item total correlations					
	Total Score		Short Form		Case Definition	

	Female	Male	Female	Male	Female	Male

Sore throat	.40	.28			.34	.30
Tender nodes	.42	.24			.37	.22
Diarrhea	.40	.47				
Unusual fatigue after exertion	.66	.82	.69	.86	.61	.84
Muscle aches	.48	.63	.44	.57	.59	.67
Joint pain	.38	.50			.43	.52
Feverishness	.35	.27				
Chills	.36	.60				
Unrefreshing sleep	.69	.83	.75	.87	.61	.82
Sleeping problems	.64	.72	.66	.78		
Headaches	.36	.64			.41	.62
Memory problems	.56	.66	.56	.64		
Concentration	.66	.86	.68	.89	.57	.78
Nausea	.49	.42				
Stomach pain	.45	.47				
Sinus problems	.27	.40				
Shortness of breath	.38	.41				
Sensitivity to light	.40	.60				
Depression	.41	.56				

### Validity

#### Convergent validity of the CDC Symptom Inventory

The Pearson's correlation coefficient indicated a good convergent validity of the CDC Total, Case Definition and Short Form scores as determined by correlations with the CIS and the SF-36 in men and women (Table 4). Severity of fatigue coincided with severity of accompanying symptoms and physical impairment.

**Table 4:** Pearson's correlation matrix of CDC Symptom Inventory DLV, CIS-20 and SF-36 scores for women and men.

	Total score		Short Form		Case Definition	
Questionnaires						
**Women (n = 127)**	r	*P*	r	*P*	r	*P*
CIS-20						
Fatigue	.64	<.001	.69	<.001	.65	<.001
Concentration	.65	<.001	.69	<.001	.67	<.001
SF-36						
Physical functioning	-.68	<.001	-.64	<.001	-.64	<.001
CDC Symptom Inventory DLV						
Total			.92	<.001	.93	<.001
Short Form	.92	<.001			.90	<.001
**Men (n = 55)**						
CIS-20						
Fatigue	.81	<.001	.82	<.001	.84	<.001
Concentration	.79	<.001	.84	<.001	.79	<.001
SF-36						
Physical functioning	-.84	<.001	-.80	<.001	-.85	<.001
CDC Symptom Inventory DLV						
Total			.96	<.001	.96	<.001
Short Form	.96	<.001			.95	<.001

#### Construct validity

**Figure 1:**
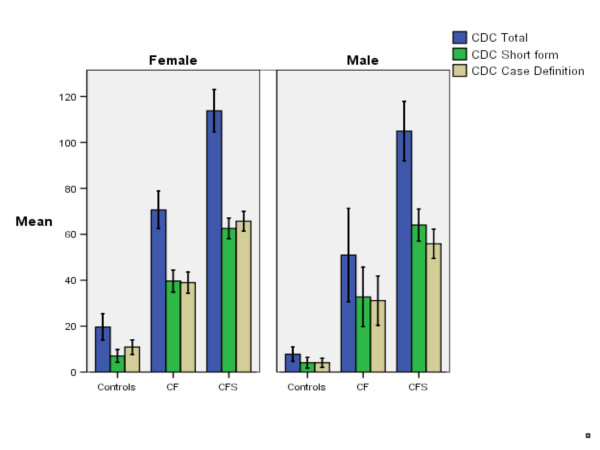
Total, Short Form and Case Definition score of the CDC Symptom Inventory – DLV for healthy controls, Chronic Fatigue patients who did not fulfill the criteria for CFS (CF) and patients with Chronic Fatigue Syndrome (CFS). Error bars indicate Standard Error of the Mean.

**Figure 2:**
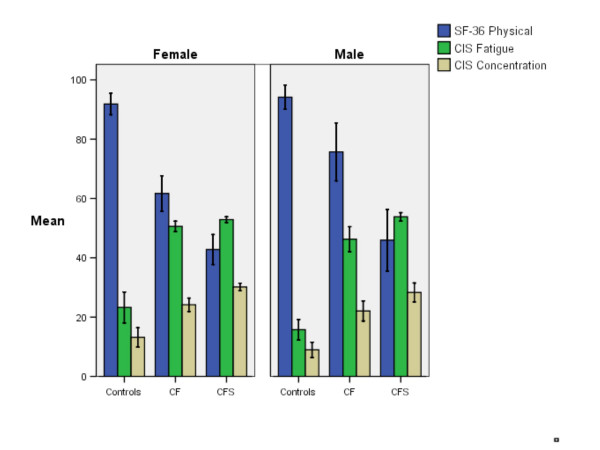
SF-36 Physical Functioning, CIS Fatigue and CIS Concentration scores for healthy controls, Chronic Fatigue patients who did not fulfill the criteria for CFS (CF) and patients with Chronic Fatigue Syndrome (CFS). Error bars indicate Standard Error of the Mean.

The Bonferroni post-hoc comparisons between healthy controls, chronic fatigue and chronic fatigue syndrome patients showed significant mean differences related to CDC Symptom Inventory scores in men and women (Figures 1 and 2) (Bonferroni post-hoc test; *P *< 0.001).

## Discussion

This evaluation of the clinical application of the Dutch translation of the CDC Symptom Inventory shows that it is a reliable tool for the assessment of CFS.

The patients differed in some respects from the population studied by Wagner et al. [6]. We analyzed consecutive patients who attended a tertiary care setting during one year and their healthy accompanying relatives and friends. The majority of CFS patients were female, the accompanying friends were male, and the relatives were most often parents, which explains the difference in the male – female ratio and age between the healthy controls and the patients. The gender difference was analyzed and proved relevant for the outcome.

The differences of the populations explain the different results of the analyses in the two studies, but the trend is comparable. The reliability of the scores, expressed as Cronbach's α coefficient was almost identical in the two studies. The relation of the outcome of the CDC Symptom Inventory (DLV) and the SF-36 Physical Functioning score was comparable. The CIS-20 Fatigue and Concentration scores in our analysis were closely related to the CDC Symptom Inventory DLV scores with correlation coefficients that were comparable to the MFI General Fatigue and Mental Fatigue scores in the study of Wagner et al. [6].

We limited the number of tests because of clinical relevance for the patients. More tests would have added little to the clinical diagnosis of CFS.

## Conclusion

The Dutch translation of the CDC Symptom Inventory proved to be a reliable tool in the clinical setting of a tertiary care center. The translated version is reliable and the results are comparable to the study of a different population in an English-speaking country. The different response of men and women to the tests warrants further study.

## Competing interests

The author(s) declare that they have no competing interests.
